# Family-school autonomy support for children’s responsible use of generative artificial intelligence: a self-determination theory synthesis and developmental research agenda

**DOI:** 10.3389/fpsyg.2026.1969217

**Published:** 2026-09-11

**Authors:** Yaoyao Fan, Xue Li, Renyuan Zhang

**Affiliations:** 1 Wuchang Institute of Technology, Wuhan, Hubei, China; 2 Jiujiang Polytechnic University of Science and Technology, Jiujiang, Jiangxi, China; 3 Institute of Student Physical Health Promotion, Jingchu University of Technology, Jiangmen, Hubei, China

**Keywords:** academic integrity, artificial intelligence literacy, autonomy support, children and adolescents, generative artificial intelligence, learning engagement, parental mediation, self-determination theory

## Abstract

Generative artificial intelligence is becoming embedded in children’s and adolescents’ learning, yet guidance often oscillates between uncritical adoption and restrictive control. This mini review applies self-determination theory to ask how autonomy-supportive guidance from families and schools might foster responsible use, and it audits how far the available evidence can carry that account. Of the 46 primary-data studies reviewed, 35 sampled university students and 8 included participants under 18; 26 were cross-sectional, and none measured family and school inputs in the same participants. Within those limits, current evidence suggests that educational outcomes depend less on frequency of use than on whether learners retain cognitive agency, engage reflectively, and regulate when and why they delegate tasks to artificial intelligence. Autonomy support may strengthen learning engagement by satisfying needs for autonomy, competence, and relatedness and by supporting intrinsic motivation, hope, self-efficacy, and self-regulated learning. It may also protect academic integrity by facilitating moral internalization, authentic self-attribution, and reasoned decision-making within clear institutional boundaries. Conversely, controlling or weakly structured environments may promote dependent cognitive offloading, unreflective use, and overreliance. Because direct evidence in minors is scarce, we treat the developmental claim as an agenda rather than a finding: we present family-side and school-side evidence separately, state their synergy as three competing and falsifiable hypotheses, and grade each proposed link by the strength of evidence behind it. We identify priorities for longitudinal, behavioral, cross-cultural, and participatory research.

## 1 Introduction

Generative artificial intelligence (GenAI), exemplified by ChatGPT, has rapidly entered young people’s learning, recreation, and everyday communication since late 2022. In a large survey, more than 60% of Chinese university students reported academic GenAI use during the 2023-2024 academic year, while naturalistic keystroke observations showed that US adolescents used AI chatbots for instrumental tasks, emotional support, and role-play (Guo et al., 2025; Maheux et al., 2026). A developmental perspective is essential because GenAI may function as a relational partner, and its meaning and risks are likely to vary with age and developmental tasks (Liu and Yip, 2026).

This mini review uses self-determination theory (SDT) to organize evidence on autonomy-supportive guidance from families and schools and its potential pathways to learning engagement and academic integrity. Here, responsible use means calibrated, transparent, and reflective engagement in which learners retain authorship, verify outputs, and match AI assistance to legitimate learning goals. Because direct evidence in minors remains limited, the review draws cautiously on university-student studies to identify provisional mechanisms without assuming developmental equivalence.

The reach of that strategy is bounded by what the literature contains. Of the 46 primary-data studies cited here, 35 sampled university students and 8 included participants under 18; 26 were cross-sectional, 4 used two or more waves, and 2 were experimental. Four measured a family input and 15 a school input, and none measured both in the same participants (Supplementary Table S1). We therefore present family-side and school-side evidence separately, state their coordination as competing hypotheses rather than as an effect, and grade each proposed link by the evidence behind it.

### 1.1 Review approach

This narrative mini review used a focused, theory-guided approach rather than a systematic-review protocol. Recent peer-reviewed empirical studies, reviews, and conceptual papers addressing GenAI use, autonomy support, AI literacy, cognitive offloading, learning engagement, or academic integrity were prioritized. Evidence concerning children, adolescents, families, and schools was emphasized. When child-specific evidence was unavailable, higher-education findings were included to identify provisional mechanisms and were interpreted cautiously rather than generalized directly to younger learners. Each cited study was then coded for sample population, design, and whether family or school inputs were measured; the coding rules and the full audit are reported in Supplementary Table S1.

## 2 Conceptualizing GenAI use: from dependence to agency

### 2.1 Use is multidimensional

Among 72,615 Chinese university students, academic use was positively associated with cognitive and emotional engagement but also with lower academic motivation and less active learning (Guo et al., 2025). A dual-pathway account distinguishes capability-oriented familiarity from exposure-oriented frequency: capability was associated with greater perceived cognitive facilitation and lower integrity-related risk, whereas frequency showed weaker and less stable associations (Mu et al., 2026). Responsible use should therefore be conceptualized through competence, behavioral exposure, purpose, and quality rather than a simple high-low continuum.

### 2.2 Dependent versus autonomous cognitive offloading

Cognitive offloading, the use of physical or digital action to reduce internal cognitive demand (Risko and Gilbert, 2016), provides a sharper account of how GenAI changes learning. A three-wave study of 589 university students and early-career knowledge workers distinguished dependent offloading, in which core thinking is delegated to AI, from autonomous offloading, in which AI provides scaffolding while the learner retains epistemic agency. Dependent offloading was associated with transferred agency, lower intrinsic motivation, and poorer perceived cognitive outcomes; autonomous offloading showed the opposite pattern (Zhu et al., 2026). Immediate benefits did not differ, making maladaptive engagement difficult to detect. Related research differentiates reflective from unreflective use: reflective use was associated with less academic impostor syndrome and stronger engagement, whereas unreflective use showed the reverse pattern (Wang et al., 2026). User profiles likewise range from constructive to substitution-oriented, with about one fifth of students in an English-as-a-foreign-language sample relying heavily on AI-generated text (Alsadoon, 2026).

### 2.3 From overreliance to problematic use

At the maladaptive end of the continuum, academic AI overreliance comprises cognitive dependence and emotion-dysregulation-related reliance. It is better understood as externalization of cognitive and self-regulatory processes than as conventional technology addiction (Oleas et al., 2026). Academic stress may operate through performance expectations, anxiety, and craving-related thinking (Liu et al., 2026); low need for cognition may operate through positive affect and avoidance-oriented motivation, with risk amplified by stress and buffered by AI literacy (Kong et al., 2026). These mechanisms identify more precise intervention targets than blanket limits on use.

## 3 Family-school autonomy support

### 3.1 Self-determination theory as an organizing framework

SDT proposes that satisfaction of the needs for autonomy, competence, and relatedness supports internalized motivation and healthy development (Ryan and Deci, 2000). In educational technology settings, need satisfaction has been associated with AI literacy, partly through cognitive engagement, metacognitive knowledge, resource management, and motivational beliefs (Wang et al., 2025). Perceived technological and teacher support also showed indirect associations with AI literacy through autonomy and competence satisfaction, although the relatedness pathway was not significant (Shen and Cui, 2024).

### 3.2 Evidence from schools: teacher and institutional support

Among vocational English learners, competence and behavioral support were indirectly associated with learning outcomes through academic self-efficacy (Xia, 2026). In fashion-design education, technical and instructional support predicted GenAI use, whereas emotional support had no direct association (Zhang, 2026). In AI-assisted language classes, AI trust and self-efficacy were associated with classroom engagement indirectly through basic need satisfaction (Guo and Zheng, 2026). A latent-profile study of German secondary students further found that perceived teacher support and ChatGPT assistance jointly differentiated motivational-emotional profiles, with teacher support the strongest predictor of an adaptive profile (Schweder, 2026).

Institutional provision matters alongside interpersonal support. AI self-study rooms were associated with gains in motivation, self-regulation, enjoyment, and engagement among primary-school learners (Li et al., 2026), while ethics guidance and coursework were associated with ethical decisions through moral cognition (Deng H. et al., 2026). Across this literature the predictors are school-side throughout: none of these studies measured what learners encountered at home.

### 3.3 Evidence from families: parental mediation

Family evidence is thinner and largely descriptive. Parental mediation theory distinguishes restrictive, active, and co-use strategies and locates their effect in the rationale conveyed rather than in the amount of restriction (Clark, 2011), and parental autonomy support has long been associated with children’s self-regulation and competence at school (Grolnick and Ryan, 1989). Interviews with 16 parents of young children across nine Chinese cities suggested a process in which parental beliefs shape AI’s assigned functions and mediation strategies for presence, exposure, screen time, and content (Zhang et al., 2026a). Access is socially patterned: 28.4% of surveyed parents of children with speech and language disorders used ChatGPT for health information, with use varying by gender, education, and participation in formal treatment (Şimşek and Güneş, 2026). In pediatric chronic-care contexts, families preferred a condition-specific chatbot to general-purpose ChatGPT, emphasizing trust and specificity (Lau et al., 2025). Independent AI use and family discussion predicted stronger endorsement of AI ethical principles among Chinese middle-school students (Zhang et al., 2026c). These four studies are qualitative, cross-sectional, or mixed-method, three sample parents rather than children, and none measured a school or teacher variable.

### 3.4 Coordination as a testable hypothesis

**Figure 1:**
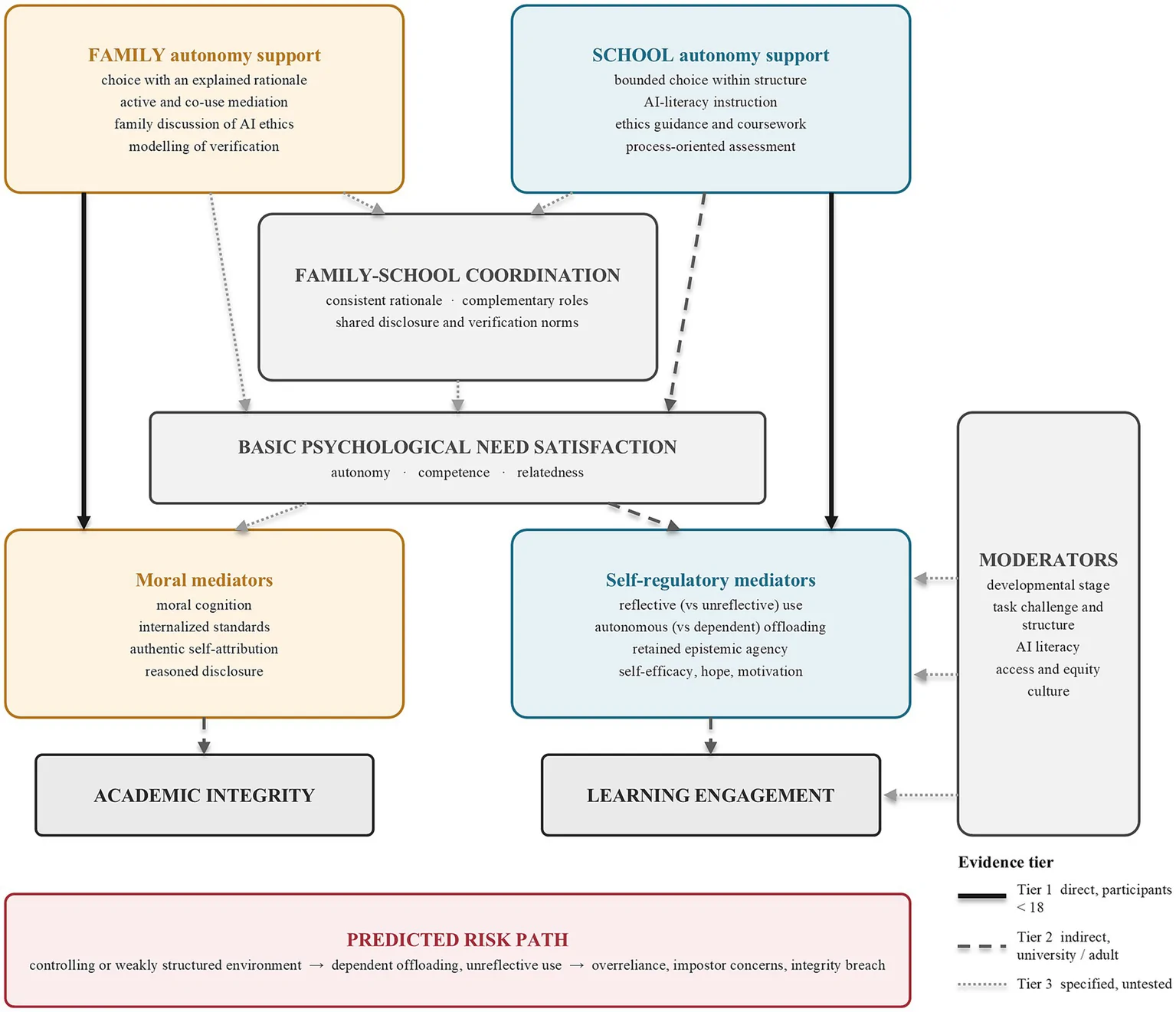
A family-school autonomy-support framework for children’s responsible use of generative artificial intelligence (GenAI), with every link labeled by the strength of the evidence currently available for it. Family-side and school-side inputs (Level 1) are hypothesized to act both directly and through a coordination layer (Level 2) on basic psychological need satisfaction (Level 3), which in turn supports moral and self-regulatory mediators (Level 4) and, through them, academic integrity and learning engagement (Level 5). Line style denotes evidence tier: Solid, Tier 1 (direct evidence in participants under 18); dashed, Tier 2 (indirect evidence from university or adult samples); dotted, Tier 3 (theoretically specified and untested). Because no reviewed study measured a family and a school input in the same participants, every link into and out of the coordination layer is Tier 3. The two Tier 1 links bypass the proposed mechanism: the only findings in minors connect a setting directly to a mediator, with no intervening measure of need satisfaction. Moderators are hypothesized to apply to all paths, and the risk path states the predicted consequence of controlling or weakly structured environments. Propositions and disconfirming results for each link are given in Table 1.

The two literatures above therefore rest on non-overlapping samples, and the coordinated model this review proposes has not been tested. Coordination remains plausible: family-school partnership interventions improve children’s social–emotional functioning in other domains, with efficacy moderated by which components are combined (Sheridan et al., 2019). No equivalent test exists for GenAI, so synergy should be treated as a hypothesis rather than an established effect, and three competing versions of it can be distinguished. H1 (additive): family and school autonomy support each predict responsible use, giving two main effects and no interaction. H2 (synergistic): the association between school autonomy support and responsible use is stronger where family autonomy support is high, giving a positive interaction. H3 (compensatory): support in either setting is sufficient, giving a negative interaction and diminishing returns when both are high. Discriminating them requires comparable autonomy-support measures obtained from the same students in both settings, a design powered for the interaction rather than for main effects, and behavioral rather than self-reported indices of responsible use; a factorial trial contrasting family-only, school-only, coordinated, and usual-practice guidance would be the strongest test. H2 is the version embedded in most current guidance and the one for which no evidence yet exists. The framework it specifies is set out in Figure 1 and Table 1.

**Table 1:** Operational matrix for the framework in Figure 1: Seven propositions with their family-side and school-side operationalizations, hypothesized processes, current evidence tier, and the result that would disconfirm each.

Proposition (Figure 1 level)	Family-side operationalization	School-side operationalization	Hypothesized process → outcome	Current evidence tier	Result that would disconfirm it
P1. Autonomy support raises autonomy satisfaction (Levels 1 → 3 → 4)	Choice over when and how GenAI is used, with the rationale for limits explained	Bounded tool choice within task structure, with task-specific rules explained	Autonomy satisfaction → autonomous motivation → reflective use	School: Tier 2 (Shen and Cui, 2024). Family: Tier 3	Autonomy support predicts use frequency but not use quality once AI literacy is controlled
P2. Competence support raises competence satisfaction (Levels 1 → 3 → 4 → 5)	Modeling of prompting, output checking, and staged withdrawal of assistance	AI-literacy instruction, source verification, process-oriented assessment, provision of AI facilities	Competence satisfaction → self-efficacy and intrinsic motivation → autonomous offloading → engagement	School → engagement: Tier 1 (Li et al., 2026; Schweder, 2026). School → needs: Tier 2 (Guo and Zheng, 2026). Family: Tier 3	Instruction raises competence but engagement gains disappear when task difficulty is held constant
P3. Relatedness sustains disclosure rather than concealment (Levels 1 → 3 → 4)	Non-evaluative discussion of outputs, emotions, and ethical dilemmas	Teacher availability and a help-seeking classroom climate	Relatedness → reasoned disclosure and help-seeking → integrity	Tier 3 both sides; the relatedness path was non-significant in the one available test (Shen and Cui, 2024)	Relatedness predicts disclosure only where sanctions are mild, indicating an instrumental rather than motivational effect
P4. Coordination as message consistency (Level 2)	The rationale for limits stated at home	The same rationale stated in course policy	Cross-setting consistency → need satisfaction over and above each setting’s main effect	Tier 3. No cited study measured a family and a school input in the same participants	The family × school interaction is null once both main effects are modeled (additive, not synergistic)
P5. Coordination as complementary role division (Level 2)	Sustains disclosure norms and everyday verification habits	Supplies verification procedures and assessment criteria	Division of labor → moral and self-regulatory mediators jointly	Tier 3	The two inputs prove substitutable, either alone reaching the same ceiling (compensation, not complementarity)
P6. Normative input is internalized rather than merely obeyed (Levels 1 → 4 → 5)	Discussion of AI ethics and of authorship expectations	Ethics guidance, ethics coursework, and explicit disclosure requirements	Internalized standards and authentic self-attribution → academic integrity	Family: Tier 1 (Zhang et al., 2026c). School: Tier 2 (Deng H. et al., 2026)	Internalized standards predict self-reported but not behaviorally recorded integrity
P7. Developmental stage moderates the relative weight of the two settings (moderator)	Mediation carries most weight in childhood	Instructional and assessment structure carries rising weight through adolescence	Age moderates the relative strength of the family and school paths	Tier 3	Multi-group models show invariant path weights across age bands

Evidence tiers: Tier 1, direct evidence in participants under 18; Tier 2, indirect evidence from university or adult samples; Tier 3, theoretically specified and untested. Tiers were assigned from a coding of the reviewed studies (Supplementary Table S1). The matrix is theory-guided rather than the product of a systematic search, and the propositions are stated so that each can be rejected by the result in the final column.

## 4 Psychological pathways to learning engagement

### 4.1 Need satisfaction, enjoyment, and motivation

Autonomy-supportive guidance may influence engagement through motivational and affective processes. In a two-wave study, attitudes toward AI were associated with engagement through a serial pathway involving perceived autonomy and learning enjoyment (Liang and Reiss, 2025). A 10 week quasi-experiment in vocational education instead identified perceived competence, but not perceived autonomy, as a mediator between AI-supported teaching and engagement (Yin et al., 2026). Thus, autonomy and competence are not interchangeable; their relative contribution is likely to depend on task structure and learner characteristics.

Intrinsic motivation and hope provide additional pathways. Satisfaction of all three psychological needs was indirectly associated with deep processing and lower overreliance through intrinsic motivation, with competence showing the strongest protective role (Fang and Feng, 2026). GenAI acceptance was associated with engagement through sequential links involving learning motivation and hope, while self-directed learning moderated the transition from motivation to hope (Zhang et al., 2026b,c. Deng C. et al., 2026).

### 4.2 Self-regulation and retained cognitive agency

Technology acceptance is educationally useful only when translated into self-regulated action. Among university students, learning motivation had a direct association with knowledge application, and autonomous technology use partly mediated this association; human-machine synergy did not add an indirect benefit (Cheng et al., 2026). Together with evidence that self-directed learning conditions motivation-to-hope pathways (Zhang et al., 2026b), these findings suggest that the central question is not whether learners use AI, but whether they plan, monitor, verify, and revise their use.

### 4.3 Cognitive costs and boundary conditions

GenAI can reduce extraneous effort while also displacing productive struggle. AI dependence was negatively associated with critical thinking partly through cognitive fatigue; information literacy buffered the critical-thinking association but, at high dependence, intensified fatigue (Tian and Zhang, 2025).

Academic self-efficacy and teacher-student relationships appear to condition these effects. Perceived AI capability was indirectly associated with engagement through learning self-efficacy, and the pathway was stronger under higher-quality teacher-student relationships (Xiang and Zhang, 2026). High self-efficacy also attenuated the negative association between AI perceptions and engagement (Shi et al., 2026). Autonomy support should therefore preserve challenge, calibrate scaffolding, and strengthen learners’ confidence in their own thinking.

## 5 Psychological pathways to academic integrity

### 5.1 Moral cognition and ethical decision-making

Academic integrity is not only compliance with rules; it requires internalized standards and context-sensitive judgment. A study integrating the stimulus-organism-response framework with social cognitive theory found that AI ethics guidance and academic ethics coursework predicted ethical decisions through moral cognition, with cognitive complexity strengthening the association between normative input and decision-making (Deng H. et al., 2026). External rules may therefore influence behavior most effectively when learners understand their rationale and can apply principles across ambiguous situations. A student-partnered Delphi study in medical education translated this logic into 14 consensus principles for responsible GenAI use (Simoni et al., 2026).

### 5.2 Self-evaluation and the student-teacher gap

Students and teachers often draw the boundary of acceptable use differently. Most surveyed health-professions graduate students regarded GenAI use in graded tests and papers as an integrity threat but considered its use for ungraded learning and review acceptable (Kazley et al., 2025). In English writing, students were more favorable than teachers and used ChatGPT for roughly one-third of writing time, mainly for editing and brainstorming; heavier copying was associated with weaker integrity beliefs (Alsadoon, 2026). Across 2,883 university students in 15 countries, attitudes, AI proficiency, and moral obligation were the strongest predictors of intended AI misconduct, while previous conventional and AI-related misconduct also mattered (Koscielniak et al., 2026).

Self-evaluative concerns complicate this boundary. Reflective use was associated with less academic impostor syndrome, whereas unreflective use was associated with more (Wang et al., 2026). Qualitative evidence identifies a central conflict between efficiency and authentic learning, with cognitive offloading and ambiguous authorship occurring together, and nursing students describe the same tension between efficiency and success earned through personal effort (Zgambo et al., 2025; Zoromba and El-Gazar, 2026). Integrity guidance must therefore address identity, authorship, and value internalization, not merely detection.

### 5.3 Policy clarity with autonomy support

Perceived policy clarity amplified the contrasting associations of reflective and unreflective use with impostor syndrome: clear rules strengthened both the protective pattern for reflection and the risk pattern for unreflective use (Wang et al., 2026). Students in nursing education nevertheless report insufficient guidance about acceptable GenAI use (Siah et al., 2026). Process-aware pedagogy, source verification, citation skills, and redesigned assessments can make standards actionable (Pireci Sejdiu et al., 2026; Peters and Angelov, 2025; Iheduru-Anderson, 2026; Astbury et al., 2026). Clarity is most likely to support integrity when rules specify acceptable assistance, explain why boundaries matter, offer practice in disclosure and verification, and preserve meaningful choice within those boundaries.

## 6 Controversies and conditional effects

### 6.1 Cognitive amplifier or cognitive substitute?

Evidence for amplification includes a three-level meta-analysis reporting a moderate overall benefit for learning outcomes (*g* = 0.499) and a larger effect for comprehension, cognition, and creativity (*g* = 0.669) (Fan et al., 2026). A meta-analysis of foreign-language emotions reported a large improvement (*g* = 0.788), especially for reducing anxiety and boredom (Chen et al., 2026).

Evidence for substitution includes associations between dependence, fatigue, and weaker critical thinking (Tian and Zhang, 2025), the coexistence of deep use and surrendered agency (Si et al., 2026), and heavy reliance on generated text among a subgroup of writers (Alsadoon, 2026). The apparent conflict partly reflects design: experiments often capture short-term performance under structured conditions, whereas surveys and qualitative studies are more sensitive to habitual reliance and authorship concerns. The dual-mechanism account is therefore more defensible than a uniformly positive or negative position (Alubthane, 2026).

### 6.2 Who uses GenAI, how, and under what conditions?

Educational context is a primary moderator. Positive associations with engagement were strongest in high-challenge, high-support classrooms, whereas use was negatively associated with engagement in low-challenge, high-support settings (Guo et al., 2025). Developmental stage, discipline, and tool-task fit may further alter outcomes, as suggested by K-12, programming, and music-education findings (Zhang H. et al., 2026; Li et al., 2026; Wu and Zhang, 2025; Peng et al., 2026).

Individual vulnerabilities also matter. Maladaptive perfectionism, neuroticism, self-critical perfectionism, impulsivity, need frustration, negative academic emotions, and academic pressure have been linked to dependence-related outcomes (Sun P. et al., 2026; Zhong et al., 2024; Fu, 2026). In language learning, gender and academic level moderated emotional responses to GenAI (Dong et al., 2026). Across these differences, use pattern remains pivotal: reflective use and autonomous offloading are associated with more adaptive outcomes, while unreflective use and dependent offloading are associated with risk (Zhu et al., 2026; Wang et al., 2026). Responsible-use guidance should therefore be differentiated rather than universal.

### 6.3 Autonomy support is not permissiveness

A false policy choice separates unrestricted autonomy from technological control. In SDT, autonomy support combines meaningful choice and acknowledged perspective with clear structure, competence support, and a non-controlling rationale; autonomy support and structure are separable dimensions, and the configuration combining both is associated with the most adaptive self-regulated learning (Vansteenkiste et al., 2012). Detection or punishment cannot by itself cultivate judgment, while encouragement without standards leaves learners uncertain about authorship. The most coherent position integrates transparent boundaries with AI literacy, reflective-use training, process-oriented assessment, and practice in disclosure and verification (Pireci Sejdiu et al., 2026; Peters and Angelov, 2025; Iheduru-Anderson, 2026).

## 7 Research gaps and future directions

### 7.1 Stronger designs and behavioral measurement

Most studies are cross-sectional and self-reported, limiting causal and developmental inference. Future work should combine preregistered longitudinal and experimental designs with behavioral traces, log files, screen recordings, and learning products. Screen-capture research in language learning and naturalistic keystroke observation among United States youth demonstrate how behavioral data can complement self-report (Maheux et al., 2026; Alsadoon, 2026). Privacy-preserving protocols are essential when participants are minors.

### 7.2 Developmental and family mechanisms

Comparative studies across early childhood, primary school, adolescence, and higher education are rare, even though developmental theory predicts different meanings and risks (Grundmeier et al., 2026; Liu and Yip, 2026). Family research remains predominantly qualitative or context-specific (Zhang et al., 2026a; Şimşek and Güneş, 2026). Studies should test how parental beliefs, mediation styles, child temperament, and school guidance interact over time, and whether alignment between home and school is additive, synergistic, or sometimes counterproductive, as specified in H1 to H3 above.

### 7.3 Measurement and curriculum development

Measures of AI literacy, dependence, and overreliance were largely developed in single-country or specialized university samples (Zhang et al., 2026; Oleas et al., 2026; Lin et al., 2026). Curriculum research should integrate AI competencies into broader digital literacy while specifying developmental progression (Altamimi and Al-Haija’a, 2026).

### 7.4 Equity, participatory design, and governance

Evidence is concentrated in China, the United States, and Europe. Rural Indian K-12 settings face infrastructure constraints, limited teacher AI literacy, language barriers, and parental skepticism (Goyal et al., 2025), while pediatric rehabilitation data show socially patterned access (Şimşek and Güneş, 2026). Only a small proportion of reviewed higher-education studies jointly address psychological and equity dimensions (Ni and Li, 2026). Participatory design can incorporate children’s perspectives and developmental needs, as suggested by pediatric AI frameworks, youth views in health care, and adolescent co-design in health communication (Radesky et al., 2026; Reinhart et al., 2026; Sun T. et al., 2026). These evidence gaps and proposed research directions are summarized in Supplementary Table S2.

## 8 Discussion

The reviewed evidence supports a conditional account of GenAI in education. Autonomy-supportive families and schools may foster responsible use by satisfying psychological needs, strengthening motivation and self-efficacy, promoting reflective self-regulation, and helping ethical standards become internalized. Controlling or weakly structured environments may instead increase dependent offloading, unreflective use, uncertainty, and overreliance. The practical goal is not minimal use, but calibrated delegation: learners should know when AI can scaffold thinking, when productive struggle should be preserved, and how assistance must be verified and disclosed.

Two limits qualify the title of this review and not merely its findings. First, 35 of the 46 primary-data studies sampled university students and only 8 included anyone under 18, so every developmental claim made here is an extrapolation whose validity is itself an open empirical question rather than a caveat attached to a settled result. Second, because no cited study measured family and school inputs in the same participants, the coordination represented in Figure 1 is a specification to be tested, not a mechanism that has been observed. The framework is therefore a testable synthesis, not an established causal model. Progress requires developmentally appropriate AI literacy curricula, longitudinal tests of home-school coordination, participatory design, and governance that joins autonomy support to transparent boundaries. This integration may help AI augment, rather than replace, human cognition while protecting engagement and academic integrity.
