# Supplementary material for: Family-school autonomy support for children’s responsible use of generative artificial intelligence: a self-determination theory synthesis and developmental research agenda
Source: Front Psychol. 2026 Sep 11;17:1969217. doi: 10.3389/fpsyg.2026.1969217 (PMC13613973; doi:10.3389/fpsyg.2026.1969217)
Supplement: Supplementary file 1 [file Supplementary_file_1.docx]

# Supplementary Material

## Supplementary Methods. Coding procedure for the evidence audit

This mini review is narrative and theory-guided; no protocol was registered, and no exhaustive database search was conducted. We applied the audit below after assembling the literature to make the evidence base behind each claim inspectable and to assign the evidence tiers shown in Figure 1 and Table 1 of the main text.

## Family input measured

Coded Yes when the study measured a variable originating with parents or the home (parental mediation, parental attitudes or rules, co-use, family discussion) as a predictor, moderator, or focal construct. Studies that discuss families without measuring a family variable were coded No.

## School input measured

Coded Yes when the study measured a variable originating with teachers, the classroom, or the institution (perceived teacher support, AI-literacy instruction, ethics guidance or coursework, assessment design, institutional policy or facilities). Studies that discuss schooling without measuring a school variable were coded No.

## Participants under 18

Coded Yes when the reported sample includes participants below 18 years of age. Studies reporting only an adult sample, or reporting no sample, were coded No.

## Both inputs in the same participants

Coded Yes only when a single study measured a family input and a school input in the same participants. No reviewed study met this criterion, which is why every link into and out of the coordination layer of Figure 1 is assigned Tier 3.

## Evidence tiers

Tier 1 was assigned to a link when at least one reviewed study tested it in participants under 18. Tier 2 was assigned when the link is supported only in university or adult samples. Tier 3 was assigned when the framework specifies the link but no reviewed study tested it.

## Limitation of the coding

Coding was based on the bibliographic record and abstract; where these did not report the sample or the measured constructs, the study was coded conservatively as not measuring the input in question. The counts should therefore be read as a lower bound on the number of studies measuring family or school inputs, and the audit is an audit of the cited literature rather than of the field as a whole.

# Supplementary Tables

Supplementary Table S1. Study-level coding of the reviewed literature (n = 61 studies). Coding was applied to population, design, country or region, and whether the study measured an input from the family, an input from the school, or included participants under 18. Counts derived from this table: 46 studies report primary data; 35 of those sampled university students and 8 included participants under 18; 26 are cross-sectional, 4 longitudinal with two or more waves, and 2 experimental or quasi-experimental; 4 measured a family input and 15 a school input; 0 measured both in the same participants. These counts support the evidence tiers in Figure 1 and Table 1.

| **Study** | **Population** | **Design** | **Country/region** | **Family input measured** | **School input measured** | **Participants under 18** |
| --- | --- | --- | --- | --- | --- | --- |
| Alsadoon (2026) | University students | Mixed methods | Saudi Arabia | No | Yes | No |
| Altamimi (2026) | No primary sample | Conceptual / commentary | not stated | No | No | No |
| Alubthane (2026) | No primary sample | Systematic review | not stated | No | No | No |
| Astbury (2026) | No primary sample | Systematic review | not stated | No | No | No |
| Chen (2026) | No primary sample | Meta-analysis | not stated | No | No | No |
| Cheng (2026) | University students | Cross-sectional survey | China | No | No | No |
| Deng (2026) | University students | Cross-sectional survey | China | No | No | No |
| Deng (2026) | University students | Cross-sectional survey | China | No | Yes | No |
| Dong (2026) | No primary sample | Systematic review | not stated | No | No | No |
| Fan (2026) | No primary sample | Meta-analysis | not stated | No | Yes | No |
| Fang (2026) | University students | Cross-sectional survey | not stated | No | No | No |
| Fu (2026) | University students | Cross-sectional survey | China | No | Yes | No |
| Goyal (2025) | Educators | Qualitative | India (Rajasthan and Delhi) | No | Yes | No |
| Grundmeier (2026) | No primary sample | Conceptual / commentary | not stated | No | No | No |
| Guo (2025) | University students | Cross-sectional survey | China | No | Yes | No |
| Guo (2026) | University students | Cross-sectional survey | China | No | Yes | No |
| Iheduru-Anderson (2026) | No primary sample | Conceptual / commentary | not stated | No | No | No |
| Kazley (2025) | University students | Cross-sectional survey | not stated | No | No | No |
| Kong (2026) | University students | Longitudinal (≥2 waves) | not stated | No | No | No |
| Koscielniak (2026) | University students | Cross-sectional survey | 15 countries | No | No | No |
| Lau (2025) | Minors and adults | Mixed methods | not stated | Yes | No | Yes |
| Li (2026) | K-12 students | Experimental / quasi-experimental | China (mainland) | No | Yes | Yes |
| Liang (2025) | University students | Cross-sectional survey | not stated | No | No | No |
| Lin (2026) | University students | Scale development / validation | not stated | No | No | No |
| Liu (2026) | No primary sample | Conceptual / commentary | not stated | No | No | No |
| Liu (2026) | University students | Cross-sectional survey | not stated | No | No | No |
| Maheux (2026) | Minors and adults | Cross-sectional survey | United States | No | No | Yes |
| Mu (2026) | University students | Cross-sectional survey | not stated | No | No | No |
| Ni (2026) | No primary sample | Systematic review | not stated | No | No | No |
| Oleas (2026) | University students | Scale development / validation | Ecuador | No | No | No |
| Peng (2026) | No primary sample | Systematic review | not stated | No | No | No |
| Peters (2025) | No primary sample | Conceptual / commentary | not stated | No | No | No |
| Pireci Sejdiu (2026) | No primary sample | Qualitative | not stated | No | No | No |
| Radesky (2026) | No primary sample | Conceptual / commentary | not stated | No | No | No |
| Reinhart (2026) | K-12 students | Qualitative | Germany | No | No | Yes |
| Schweder (2026) | Adolescents 13-17 | Cross-sectional survey | Germany | No | Yes | Yes |
| Shen (2024) | University students | Cross-sectional survey | not stated | No | Yes | No |
| Shi (2026) | University students | Cross-sectional survey | China | No | No | No |
| Si (2026) | University students | Cross-sectional survey | China | No | No | No |
| Siah (2026) | University students | Qualitative | not stated | No | No | No |
| Simoni (2026) | University students | Qualitative | not stated | No | No | No |
| Simoni (2025) | No primary sample | Systematic review | not stated | No | No | No |
| Sun (2026) | University students | Cross-sectional survey | not stated | No | Yes | No |
| Sun (2026) | Minors and adults | Qualitative | Australia | No | No | Yes |
| Tian (2025) | University students | Cross-sectional survey | China | No | No | No |
| Wang (2025) | University students | Cross-sectional survey | not stated | No | No | No |
| Wang (2026) | University students | Longitudinal (≥2 waves) | China | No | Yes | No |
| Wu (2025) | K-12 students | Cross-sectional survey | not stated | No | No | Yes |
| Xia (2026) | University students | Cross-sectional survey | China | No | Yes | No |
| Xiang (2026) | University students | Longitudinal (≥2 waves) | China | No | Yes | No |
| Yin (2026) | University students | Experimental / quasi-experimental | not stated | No | Yes | No |
| Zgambo (2025) | University students | Mixed methods | not stated | No | No | No |
| Zhang (2026) | University students | Scale development / validation | not stated | No | No | No |
| Zhang (2026b) | University students | Cross-sectional survey | China | No | No | No |
| Zhang (2026c) | Adolescents 13-17 | Mixed methods | China | Yes | No | Yes |
| Zhang (2026a) | Parents / adults | Qualitative | China | Yes | No | No |
| Zhang (2026) | University students | Cross-sectional survey | China | No | Yes | No |
| Zhong (2024) | University students | Cross-sectional survey | China | No | No | No |
| Zhu (2026) | University students | Longitudinal (≥2 waves) | not stated | No | No | No |
| Zoromba (2026) | University students | Qualitative | not stated | No | No | No |
| Şimşek (2026) | Parents / adults | Cross-sectional survey | Türkiye | Yes | No | No |

Supplementary Table S2. Evidence gaps and research priorities for family-school guidance on children's responsible use of generative artificial intelligence. This table appeared as Table 1 in the previous version and is reproduced here unchanged; the main text now carries a single display table (Table 1) giving the operational matrix for the framework.

| **Research priority** | **Current limitation** | **Recommended approach and key outcomes** |
| --- | --- | --- |
| Causal and temporal inference | Most studies are cross-sectional and self-reported; available longitudinal studies have short follow-up periods. | Use preregistered longitudinal and experimental designs with repeated assessments. Combine psychological outcomes with learning products and observed integrity behavior. |
| Developmental specificity | Evidence is concentrated in higher education, with few direct comparisons across early childhood, primary school, and adolescence. | Develop age-appropriate tasks and measures, compare developmental stages, and assess need satisfaction, cognitive agency, safety, and privacy. |
| Family-school coordination | No cited study directly tests an aligned home-school model or establishes whether coordination is beneficial. | Compare family-only, school-only, and coordinated guidance over time, and test whether alignment is additive, synergistic, neutral, or counterproductive. |
| Behavioral and process measurement | Self-report dominates, limiting assessment of actual use, delegation, verification, disclosure, and authorship. | Integrate logs, screen recordings, prompt and revision traces, and learning products. Distinguish reflective from unreflective use and autonomous from dependent offloading. |
| Measurement and curriculum evaluation | Measures are often developed in single-country or specialized university samples, and curriculum studies rarely test long-term behavior. | Validate age-appropriate measures cross-culturally and test measurement invariance. Evaluate sustained effects of AI literacy curricula on behavior, learning, critical thinking, and integrity. |
| Equity, participation, and governance | Rural, low-resource, and disability contexts are underrepresented, and children's perspectives are seldom embedded in design. | Use inclusive sampling and participatory co-design. Assess access, language and infrastructure barriers, fairness, privacy, accountability, acceptability, and child-rights safeguards. |
